# Synthesis and Properties of Macrocyclic Butanoic Acid Conjugates as a Promising Delivery Formulation for the Nutrition of Colon

**DOI:** 10.1155/2013/914234

**Published:** 2013-09-18

**Authors:** Jingui Cheng, Benpeng Li, Peipei Ma, Mengying Liu, Zhizhong Wang

**Affiliations:** ^1^Department of Chemical Engineering, Shihezi University, Shihezi 832003, China; ^2^School of Pharmacy, Ningxia Medical University, Yinchuan 750004, China

## Abstract

Butanoic acid plays a significant role in the maintenance of mucosal health and is the preferred energy substrate for the cells in the colon. Here, butanoic acid was selectively conjugated to the secondary hydroxyl group of **β**-cyclodextrin through ester bond using sodium hydride as the deprotonation reagent. The preliminary release behaviors of butanoic acid in rat gastrointestinal tract contents were investigated at 37°C within 12 h. In the contents of stomach, the conjugates did seldom release butanoic acid, released butanoic acid only 5.8% in the contents of small intestine, and released butanoic acid significantly up to 38.4% in the contents of colon. These results indicate that the conjugate activation took place site specifically in the rat colonic contents, via the biodegradation by glycosidases and hydrolases in the colon. Therefore, the **β**-cyclodextrin conjugates of butanoic acid may be of value as an orally administered colon-specific formulation for the nutrition of colon.

## 1. Introduction

Short-chain fatty acids (SCFA) are important components of nutrition and have attracted considerable interest in human health as a result of the realization that SCFA represent an important mechanism for carbohydrate and calorie conservation [1, 2]. Butanoic acid (BA), an important member of SCFA, is thought to play a significant role in the maintenance of mucosal health and is the preferred energy substrate for the cells in the colon [3, 4]. However, butanoic acid is liquid and has an unpleasant smell and acrid taste. Butanoic acid enemas smell bad, and patients are not always willing to undergo the treatment. Another problem with enemas is that the butanoic acid does not stay in the colon for very long [5]. Thus, a colon-specific delivery system of butanoic acid is expected to be a promising formulation for the nutrition of colon.

**Figure 1:**
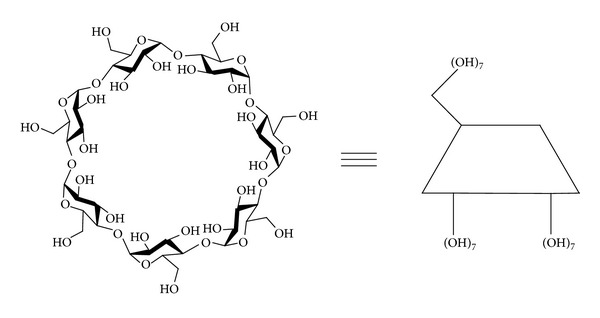
The schematic structure of **β**-CyD.

**β**-Cyclodextrin (**β**-CyD) is a well-known macrocyclic oligosaccharide consisting of 7 **α**-1, 4-linked D-glucopyranose units (Figure 1) and has been widely used as an excipient in the pharmaceutical industry for improving some properties of drugs, such as solubility, stability, absorption, and/or bioavailability, by forming the inclusion complexes [6]. On the other hand, **β**-CyD is hardly hydrolyzed and only slightly absorbed through the stomach and small intestine. However, **β**-CyD can be fermented into small saccharides by colonic microflora. This biodegradable property makes **β**-CyD useful as a colon-specific material [7]. Therefore, **β**-CyD's conjugates, where a drug is covalently bonded to **β**-CyD, may serve as a source of colon-specific delivery system of drugs. In recent years, several drug/**β**-CyD conjugates and their pharmaceutical properties have been reported [8–14].

 Here we report the preparation of BA/**β**-CyD conjugates, attempting to construct a colon-specific delivery for BA as a nutrient. The preliminary release behaviors of BA in rat gastrointestinal tract contents were investigated.

## 2. Experimental

### 2.1. Materials

**β**-CyD was recrystallized twice from distilled water and dried under reduced pressure at 110°C for 24 h before use. *N,N*-dimethylformamide (DMF) was freshly distilled over CaH_2_ and stored over 4A molecular sieves. Dichloromethane (DCM) was dried by CaCl_2_ for 12 h and distilled prior to use. All other chemical materials and reagents were of commercial grade, and directly used.

### 2.2. Analytical Methods

NMR spectra were recorded on Bruker AM-600 spectrometer (^13^C NMR, 150 MHz) in D_2_O solutions with (tetramethylsilane) TMS as standard. The ESI-MS experiment was performed using a ThermoQuest Finnigan LCQ^DECA^ system equipped with an ESI source (ThermoQuest LC/MS Division, San Jose, CA, USA). The HPLC assays were performed on a Perkin-Elmer Series 200 HPLC system using a Kromasil 100-10-C18 column (4.6 mm × 250 mm); flow rate: 1.0 cm^3^/min; detection wavelength: 220 nm; the mobile phase: methanol—0.05 M phosphate buffer (pH 2.0, 20 : 80 v/v).

### 2.3. Synthesis of the BA/*β*-CyD Conjugates

To a solution of butanoic acid (0.18 g, 2.04 mmol) in 20 cm^3^ DCM, oxalyl chloride (0.55 cm^3^) was added at room temperature. After the addition of three drops of dry DMF, the mixture was stirred overnight with a reflux condenser. After completion of the reaction, the excess oxalyl chloride was removed under reduced pressure. Thus, the crude butanoyl chloride was obtained and dissolved in DMF (5.0 cm^3^), which was used in the next step.

 NaH (60% in mineral oil, 82 mg, and 2.03 mmol) was added to a solution of **β**-CyD (2.3 g, 2.03 mmol) in DMF (100 cm^3^) at 0°C, and the mixture solution was stirred overnight. The above butanoyl chloride in DMF was added, and the mixture was stirred while allowing it to stand at room temperature for 8 h. It was evaporated under reduced pressure to a volume of ca. 5 cm^3^, and acetone (300 cm^3^) was added to precipitate the unreacted **β**-CyD and its derivatives. The precipitate was filtered and washed with acetone (80 cm^3^). The crude products were isolated by an open RP-18 column using H_2_O-MeOH (10%-20%-40%-60%-80%) as eluents. Thus, **1** was obtained in 24% yields (0.51 g). ESI-MS: *m/z* = 1227 ([M + Na]^+^); ^13^C NMR (150 MHz, D_2_O): **δ** = 13.9, 19.0, 36.7, 60.1–60.5, 70.2, 71.4–73.9, 74.7, 78.6, 81.5–82.3, 97.5, 102.3–102.7, 171.6.

### 2.4. Hydrolysis of the BA/*β*-CyD Conjugates Incubated with the Gastrointestinal Tract Contents of Rats

The hydrolysis behaviors, incubated with the gastrointestinal tract contents of male Kunming rats, were performed at 37°C according to a literature procedure [14]; that is, male Kunming rats (200 ± 10 g) were anesthetized by diethyl ether, and midline incisions were made. Contents of stomach, small intestine, and colon were collected separately, and the contents were diluted to half concentration with isotonic acetate buffer (pH 4.4) for stomach contents and with isotonic phosphate buffer (pH 6.8) for other contents, and the dispersions of contents were filtered through a gauze to remove large particles. The conjugate solution (20.0 cm^3^, 8.0 × 10^−3 ^M in the corresponding isotonic buffer) was added to the filtrate (10.0 cm^3^) in air-tight vessels and incubated at 37°C. The pH of incubation solutions was adjusted to 4.4 (stomach contents) or 6.8 (other contents) by the addition of small amounts of 0.1 M NaOH. Every one or two hours, an aliquot (1.0 cm^3^) of the reaction solution was adjusted to pH 2.0 by the addition of 1.0 M HCl, and BA was extracted out by diethyl ether (3 × 3.0 cm^3^). Then, the combined organic phases were evaporated under reduced pressure, and the residue was dissolved in methanol (0.1 cm^3^). The concentration of BA was determined by HPLC.

## 3. Result and Discussion

### 3.1. Chemistry

**Scheme 1:**
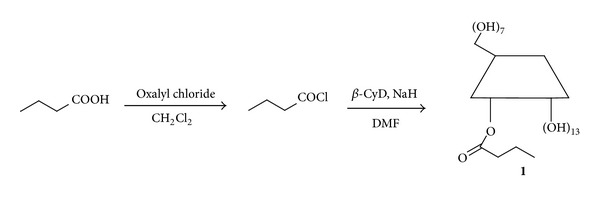
The synthesis routes of the BA/**β**-CyD conjugates.

**β**-CyD possesses the C-2 and C-3 hydroxyl groups on the secondary face and the C-6 hydroxyl groups on the primary face. Of the three types of hydroxyl groups, those at the 6th position are the most basic and often most nucleophilic, those at the 2nd position are the most acidic, and those at the 3rd position are the most inaccessible. D'Souza developed a convenient method for functionalization of the 2nd position of cyclodextrins with sodium hydride [15]. Thus, the BA/**β**-CyD conjugates were prepared in two steps as shown in Scheme 1. In the first step, butanoyl chloride was prepared using oxalyl chloride as a chlorinating agent. In the second step, the coupling of BA to **β**-CyD was accomplished in basic media using NaH as the deprotonation reagent. Therefore, BA was bonded to **β**-CyD through ester linkage.

 The structure of **1** was characterized by ESI-MS and NMR spectra. Its ESI-MS spectrum exhibited the molecular ion [M + Na]^+^ at *m/z* 1227, which indicates that the degree of substitution in the conjugate was monosubstituent BA. The ^1^H NMR spectra of **β**-CyD are complex and are not generally used to clarify the position of substituent. However, the ^13^C NMR spectrum is an effective technique for the analysis of cyclic oligosaccharides [15, 16]. Here, the ^13^C NMR of **1** demonstrated a chemical shift at **δ** 78.6 ppm (C-2′), which clearly indicates that the substituent BA is at the 2nd position of **β**-CyD.

### 3.2. Hydrolysis of the Conjugates in Rat Gastrointestinal Tract Contents

**Figure 2:**
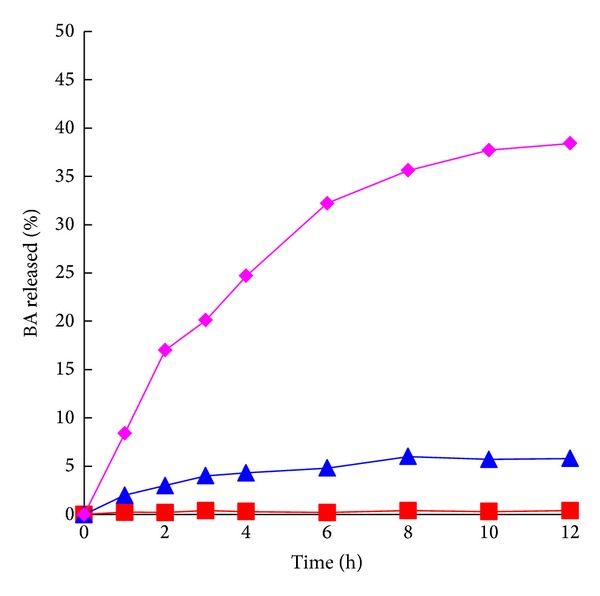
The release behaviors of BA from the BA/**β**-CyD conjugates incubated with rat gastrointestinal tract contents (17%, w/v). (1) Stomach contents (■); (2) small intestine contents (▲); (3) colonic contents (♦) at 37°C.

The preliminary release behaviors were studied for the BA/**β**-CyD conjugates at 37°C within 12 h, and the results are shown in Figure 2. It was indicated that the BA/**β**-CyD conjugates did seldom release BA in the contents of stomach, released BA only 5.8% in the contents of small intestine, and released BA significantly up to 38.4% in the contents of colon. The ESI-MS spectra of incubation solution in the contents of colon indicate that the BA/**β**-CyD conjugates were fermented into BA, BA-small saccharide conjugates, small saccharides, and glucoses. On the other hand, the release rate of BA was relatively slow, and this indicates that BA could stay in the colon for very long, while an enema of BA could not do so [5]. These results indicate that the conjugate activation took place site-specifically in the rat colonic contents, via the biodegradation by glycosidases and hydrolases in the colon [14].

## 4. Conclusion

Colonic delivery system can be achieved with carriers by making conjugates that survive passage through stomach and small intestine, but active moiety is released by enzymes specifically produced in colon. Butanoic acid was covalently bonded to **β**-CyD through ester linkage. In the contents of stomach, the conjugates did seldom release butanoic acid, released butanoic acid only 5.8% in the contents of small intestine, and released butanoic acid significantly up to 38.4% in the contents of colon. In addition, the release rate of butanoic acid was relatively slow, and it could stay in the colon for very long. These facts demonstrate that the biodegradable butanoic acid/**β**-CyD conjugates may be of value as an orally administered colon-specific formulation for the nutrition of colon.
